# The Intriguing Diagnosis of Lung Cancer in a Psychiatry Inpatient Unit: A Reflection Through a Case Report

**DOI:** 10.7759/cureus.29450

**Published:** 2022-09-22

**Authors:** Odete Nombora, Ana Miguel, Lucas Lopes, Ângela Venâncio

**Affiliations:** 1 Psychiatry Department, Vila Nova de Gaia/Espinho Hospital Centre, Vila Nova de Gaia, PRT

**Keywords:** brain metastasis, lung cancer, behavioural changes, neurodevelopmental disorders, intellectual disability

## Abstract

People with intellectual developmental disorders are vulnerable to somatic and mental illnesses, often presenting with behavioural changes. Through an intriguing and uncommon case report, we aim to provide an overview of behavioural changes in patients with an intellectual developmental disorder, emphasizing the need for screening for non-psychiatric conditions. We present a clinical case of a 57-year-old man with a personal history of intellectual developmental disorder, epilepsy, and alcohol and tobacco abuse. He had a previous acute psychiatric admission in 2017 due to behaviour disorganization and irritability. In April 2019, he was readmitted with disorganized behaviour and caregiver exhaustion. On the 58th day of hospitalization, he fell off his bed and suffered a mild traumatic brain injury. A cerebral CT scan revealed two metastatic lesions in the brain. Further investigations discovered a primary neoplastic lung lesion with metastasis to pulmonary lymph nodes. This case emphasizes that despite a long follow-up with psychiatry services, physical illness should be considered when patients with intellectual developmental disorders present with behavioural changes as they can precede image and laboratory findings. Additionally, further studies are needed in order to provide guidelines and proper medical and psychosocial care for this particular population and the caregivers.

## Introduction

This case report was previously presented as a meeting abstract and e-poster at the 2021 European Psychiatry Congress on April 10-13, 2021.

Intellectual developmental disorder, commonly known as intellectual disability, refers to a neurodevelopmental disorder characterised by significant impairments in intellectual and adaptive functioning, including daily activities and communication skills, with onset in childhood [1, 2]. In this article, the authors prefer to use the term "intellectual disability" for practical purposes.

People with intellectual disabilities account for approximately 1-2% of the general population [1-3]. Intellectually disabled people are vulnerable to organic and mental illnesses [1, 4, 5], often presenting with behavioural changes [6]. In fact, they seem to have higher rates of poor physical and mental health, and unhealthy lifestyles [1, 2, 4], which can lead to lower life expectancy and higher mortality rates compared with the general population [1, 2]. The difficulties of the patients in describing symptoms can limit their access to health care and to adequate treatment [1, 2, 4, 7]. Furthermore, this population often experience more psychosocial issues such as poverty, violence, discrimination and unemployment, reflecting the social inequalities compared to their non-disabled peers [1, 5].

Through an intriguing and uncommon case report, we aim to promote clinical awareness of behavioural changes in people with intellectual disabilities due to non-psychiatric conditions, emphasizing the relevance of the screening and assessment.

## Case presentation

We describe a clinical case of a 57-year-old man with a personal history of intellectual disability, epilepsy, and alcohol and tobacco abuse. He was single and was living with his younger brother and sister-in-law, but the family dynamic was neither comprehensive nor contained. He grew up as a farmer, did not complete the first grade due to learning difficulties and always had limitations expressing his thoughts and feelings, which lead to a poor understanding of his needs.

He had been in psychiatric outpatient care since 2014 due to his intellectual disability. In 2016, he started evidencing loss of previously existing cognitive functions with functional impact. He also exhibited changes in behaviour, such as oppositional behaviours and lack of collaboration in care, as well as agitation and aggressiveness towards his family members and caretakers when contradicted. Thus, in 2016, he was evaluated by neurology for a more detailed diagnostic screening. He also underwent an MRI that did not demonstrate structural lesions associated with the clinical condition. Neurosyphilis hypothesis and pulmonary and urinary infections were excluded, as well as thyroid disease, vitamin B12 deficiency and HIV. At the end of the same year, he was admitted to the stroke unit due to suspicion of left insular ischemic stroke, but it was not confirmed. In 2017, he began to have epileptic seizures and started valproic acid 500mg twice daily, while maintaining the psychiatric follow-up. In the same year, the behavioural changes became worse, he started being more aggressive towards his family and evidenced disorganized behaviour, as well as major irritability. Therefore, he had his first acute psychiatric admission and underwent one more cerebral CT scan, which was normal.

**Figure 1:**
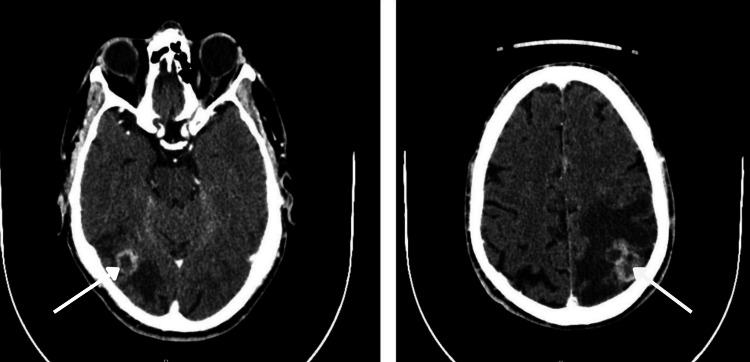
Cerebral CT scan images showing right posterior temporal (maximum diameter 17 mm) and left parietal (maximum diameter 29 mm) expansive lesions.

**Figure 2:**
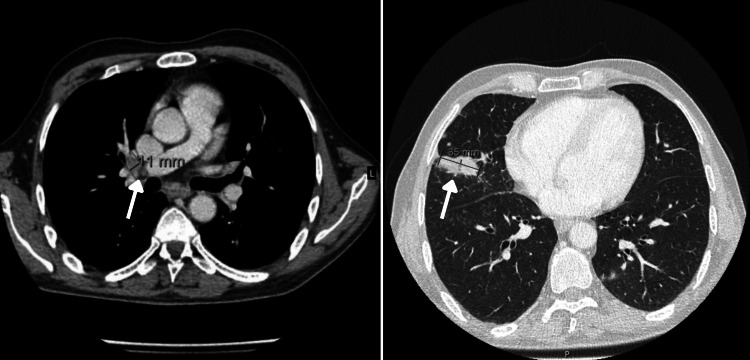
Thoraco-abdominal CT scan revealing pulmonary lymphadenopathy (left) and a lung cancer lesion (right)

In April 2019 he was again hospitalized in a psychiatric unit with the same symptomatology and additional subjective posture instability. During the second week of hospitalization, he was stabilized with 20mg/day of olanzapine. His caregiver was exhausted and unable to take care of him at home. On the 58th day of hospitalization, he fell off his bed when going to the bathroom, without loss of consciousness. However, because of his limitations in the communication of symptoms and complaints, he was again submitted to a cerebral CT scan, an electroencephalogram (EEG) and an electrocardiogram (ECG). The EEG and ECG were normal. The cerebral CT scan revealed two metastatic lesions in the brain, one in the posterior right temporal and another in the parietal lobe (Figure 1). He was then oriented for further investigations. He performed a thoraco-abdominal CT scan which showed lesions compatible with lung cancer and metastasis to pulmonary lymph nodes (Figure 2). The CT-guided lung biopsy of the lung lesions confirmed the diagnosis of lung adenocarcinoma ALK+.

When he was informed about the diagnosis, he just asked if it was severe, with no mood or behavioural changes. Weeks later, he became more anxious, with some restlessness that was easily managed with benzodiazepines.

After being informed about the diagnosis, his relatives mentioned a heavy familiar history of cancer (sisters, brother and father) and that he had postural instability signs previously to the psychiatric admission that they did not value and so did not disclose to the medical team.

The patient was then transferred to the pneumology inpatient unit for a continuation of care and further investigation. He initiated treatment with 150mg of alectinib and 16mg/day of dexamethasone, with good tolerance as well as clinical and radiological improvements. As the patient family did not have the capacity to attend to him at home, he was integrated into a senior residential institution.

## Discussion

Our case highlights the poorer health of individuals with intellectual disabilities when compared with their non-disabled peers. Because of the cognitive deficit and the lack of communication skills our patient was unable to express his needs or understand some abnormal physical sensations and symptoms.

According to the literature, individuals with intellectual disabilities also have difficulties in understanding the illness and treatment [8], just like our patient, particularly when he was informed about the cancer diagnosis.

Studies also show that, the more severe the disability, the less clear the symptomatology, which adds some diagnostic challenges and several biases [1, 3, 6]. In fact, individuals with intellectual disability may present with mood and behaviour modifications in several circumstances such as physical pain, organic and psychiatric conditions or environmental changes [6, 8, 9]. Consequently, an accurate symptomatology description is crucial to screen these conditions and should also include triggers and patterns of escalation [6].

Taking into account the diagnosis of our patient it is important to point out some characteristics of cancer in individuals with intellectual disabilities. 

The prevalence rates of cancer in individuals with intellectual disabilities are not clearly known [8, 10, 11]. Some studies suggest increasing prevalence [3, 8], but others suggest the opposite [5, 10, 11]. Assuming that people with intellectual disabilities may have unhealthy lifestyles such as smoking, alcohol consumption and poorer eating habits [11], difficulties in accessing health care and are generally polymedicated [2, 4, 5,11], it can be assumed that the risk of chronic diseases, including cancer, is increased. On the other hand, the symptoms are frequently hidden by the mental state and communication impairments, as people with this condition may not appear to express symptoms at all [3, 11], which can lead to a delay in diagnosis and treatment [3, 9, 11], as also observed in our patient.

Although lung cancer seems to be less common in people with intellectual disabilities, the possibility of lung malignancy should be considered, particularly in persons with tobacco abuse [8, 10].

Our patient had no specific physical complaints but he had risk factors for cancer such as family history as well as alcohol and tobacco abuse, both common comorbidities in intellectually disabled people [1]. As people with intellectual disabilities communicate pain and discomfort in an unconventional way, it may not be promptly understood and cancer may remain underdiagnosed for long periods of time [8, 10].

According to the timeline of our patient symptomatology, we can hypothesize that cancer appears between 2014 and 2016, as he began to evidence cognitive decline at this time, with behavioural changes posteriorly associated and epileptic seizures, despite normal thoracic radiography and cerebral images at the time. This emphasizes the importance of an in-depth assessment of these patients and that behavioural changes can precede image and laboratory findings.

Unfortunately, the behavioural changes are usually interpreted as a result of intellectual disability [3, 6, 12], as was the case with our patient. Therefore, in order to overcome diagnostic difficulties in the differential between mental/organic illnesses in these patients, it is important to remain attentive not only to the clinical presentation but also to the family history and the patient’s complaints and functionality [6]. Furthermore, it is crucial to consider the caregiver and family reports on the patient and provide understandable information, adapted for this particular population [6, 8, 12].

## Conclusions

Although psychiatric disorders are common in patients with intellectual disabilities, physicians, especially psychiatrists, should remember that behavioural changes can mask the presentation of non-psychiatric illness. Therefore, these conditions should be considered when patients with intellectual disabilities present with behavioural changes, and proper screening should be performed considering the common causes of behavioural changes and the patient’s comorbidities.

Unfortunately, our literature search revealed a lack of studies on this topic. Looking forward, this emphasizes the need for further research regarding the assessment of patients with intellectual disabilities, as well as the creation of guidelines on the medical and psychosocial care of these patients and their caregivers. Further studies should explore the participation of people with intellectual disabilities in prevention and screening health programs to obtain a clearer comprehension of this population's needs and strategies in order to tackle population-specific barriers to seeking preventive health care which is crucial to cancer prevention and early detection, such as promoting carer education on the importance of preventive services, regular physician contact and adherence to cancer screenings. Additionally, longitudinal studies examining incidence rates of cancer in people with intellectual disability and preventable risk factors for cancer development would help identify causal links and understand their course.
